# Structural Relationship between Physical Self-Concept, Occupational Instability, and Retirement Intention among South Korean Minor League Baseball Players

**DOI:** 10.3390/healthcare9050595

**Published:** 2021-05-17

**Authors:** Seungman Lee

**Affiliations:** Department of Physical Education, College of Education, Korea University, Seoul 02841, Korea; lsm14pe@korea.ac.kr; Tel.: +82-2-358-0783; Fax: +82-2-358-0866

**Keywords:** occupational instability, physical self-concept, professional minor league baseball player, retirement intention, structural relationship

## Abstract

This study aimed to verify the structural relationship between physical self-concept, occupational instability, and retirement intention among South Korean minor league baseball players. Snowball sampling was used to recruit 180 minor league players who belonged to the South Korean professional baseball team and were active as of December 2020; data were collected through an online survey. Frequency analysis, psychometric validation, descriptive statistical analysis, and path analysis were performed. The results revealed that for these participants, (1) physical self-concept had a significant negative effect on their occupational instability; (2) physical self-concept did not directly affect their retirement intention; and (3) occupational instability had a significant positive effect on their retirement intentions. These results suggest the need to devise a plan addressing the practical difficulties experienced by professional minor league baseball players and improve the physical self-concept of these players to adapt to involuntary retirement. Additionally, a supportive environment should be created to reduce mental health risks.

## 1. Introduction

South Korean professional baseball has been the most popular sport in South Korea since its introduction in 1982. It is particularly garnering the love of an increasing number of fans owing to South Korea’s international achievements in baseball, such as securing a gold medal at the 2008 Beijing Olympics. Owing to its popularity, several players aim to accomplish their dreams of becoming a professional baseball player with outstanding skills.

In the 2021 Korean Professional Baseball 2nd Rookie Draft held on 21 September 2020, 100 players were selected by a professional team. A total of 1133 people applied for the draft, which included 856 prospective high school graduates, 259 college graduates, and eight foreign amateur and professional athletes; however, approximately 9.7% of the players were selected, including 10 nominees from the first draft. Moreover, even if these players become professional players surpassing all hardships, the probability of becoming high-salaried players, through free agency, is relatively low. Furthermore, despite being nominated for a professional team, several players disappear without ever reaching the first team. Minor league players battle several hardships to survive the fierce competition and establish their position; however, most of them would give up baseball if they suspected that their careers would end in the second-team stage [1]. Nevertheless, there are many players who progress from the second team to the first team; they struggle and do not give up despite empty grounds with no cheers.

Most of the drafted rookie players are enrolled in the first team after their skills are recognized through certain accomplishments in the second team. However, there may be cases, where rookie players are directly enrolled in first teams, due to their potential exceeding that of the existing first-team players. Therefore, this process of enrollment is essential for players to ensure that they improve their skills to enter the first team, while experiencing various difficulties in the second team. Moreover, players gradually recognize the reality and plan retirement if their duration of playing in the second team is prolonged.

Retirement intention is the suppressed desire to achieve, which arises from the mental preference of doing something else [2]. Players will make the decision to retire or continue to play at the crossroads of retirement. Sports players have an earlier retirement age than other professionals. They will particularly be cornered into retirement, by their own will or others’ intentions, regardless of their age, if they decide that they cannot survive on the professional stage. Athletes retire owing to their need to support themselves through other jobs, and their retirement is motivated by an opportunity to advance their sports career or by a realistic judgment of their sports skills [3]. Thus, players on the second team inevitably debate retirement owing to uncertainty about the future.

Retirement can be equated with “desocialization” within the “sports socialization process”. Retirement and desocialization have been widely researched in sports sociology. Shim et al. [4] have reported the resocialization and desocialization processes among independent baseball players, while Park et al. [5] have studied mid-term retirement and career change in baseball players. Wi et al. [6] have reported the causes of retirement, retirement preparation factors of athletes, resources available after retirement, adaptation to retirement, and prevention and intervention for post-retirement stress, depression, tardiness, and others, by applying the conceptual model of adaptation to retirement, developed by Taylor and Ogilvie [7]. Additionally, Lee [8] has conducted a comprehensive study on the sports socialization processes of sports players.

This study has also examined the effect of physical self-concept on retirement intention. Physical self-concept refers to the degree of attitudes, beliefs, and feelings about oneself formed by interactions with other important people in terms of personal health, physical appearance, and ability [9]. Monthuy-Blanc et al. [10] and Kim et al. [11] have reported that physical self-concept is higher among excellent athletes than others. Several other studies have reported on the relationship between physical self-concept and various physical abilities and other variables [12,13].

Furthermore, this study has also investigated the mediating role of occupational instability in the relationship between physical self-concept and retirement intention. Occupational instability refers to the sense of helplessness experienced when threats of losing the job or change in the subjective details of the job are perceived, despite the employee’s desire for job stability [14]. Occupational instability has garnered increased research attention owing to the accumulated experiences of overall job insecurity, which acts as a stressor caused by job uncertainty [15,16]. According to Chung and Park [17] and Han [18], physical self-concept affects occupational instability. Additionally, previous studies suggest that accumulated job insecurity inevitably leads to retirement [19,20]. These existing study findings can predict the relationship between physical self-concept, occupational instability, and retirement intention.

Several studies have also been conducted on professional baseball players in the minor league. Kim and Ha [1] have conducted a phenomenological analysis on the projected image vs. the reality of a professional baseball player in a minor league. Additionally, Jeon et al. [21] has studied the retirement experience of professional baseball players and their social adaptation methods post-retirement, while Park [22] has investigated the obstacles and countermeasures affecting the performance of second-team professional baseball players. Moreover, Park et al. [5] has also examined mid-term retirement and career change among baseball players who retired involuntarily because they were not nominated as a professional player.

Therefore, existing literature has provided abundant meaningful insights into the reality of professional baseball players in the second team. However, most of these studies used qualitative research methods; thus, the literature lacks quantitative research studies. Additionally, there is no existing research that comprehensively examines the structural relationship between physical self-concept, occupational instability, and retirement intentions among minor league players. Therefore, this study is necessary to present the difficulties and poor treatment experienced by second-team players compared to those in popular professional baseball first-teams.

Thus, this study aimed to determine the structural relationship between physical self-concept, occupational instability, and retirement intention among professional baseball players in minor leagues. For this purpose, we hypothesized as follows:

**Hypothesis 1** **(H1).** *The physical self-concept of minor league players will negatively affect occupational instability*.

**Hypothesis 2** **(H2).** *The physical self-concept of minor league players will positively affect their retirement intention*.

**Hypothesis 3** **(H3).** *The occupational instability of minor league players will negatively affect their retirement intention*.

**Figure 1:**
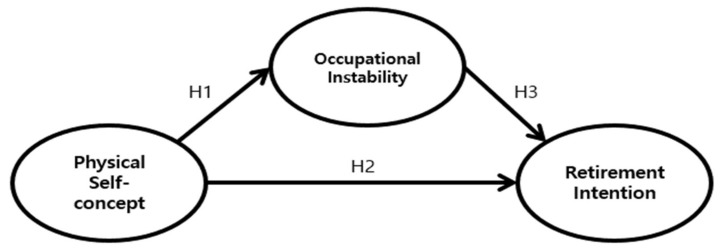
Hypothesized study model.

Figure 1 presents the hypothetical model of this study.

## 2. Materials and Methods

### 2.1. Participants

A total of 200 professional baseball players were recruited as participants in this study, using the snowball sampling method. Participants included in this study were those who: (1) were only in the minor league and never progressed to a major league, (2) belonged to and were active in the Korea Professional Baseball (KBO) club, and (3) had participated in the Korean minor league game as of December 2020. An electronic survey was conducted using Google forms, due to the government’s social distancing norms amidst COVID-19. Of these, 20 questionnaires were excluded due to missing or unscrupulous responses and 180 questionnaires were finally used for analysis. Table 1 presents the distribution of participants. This study was approved by the Institutional Review Board of Kyung Hee University, Suwon, South Korea (KHGIRB-20-658).

### 2.2. Instruments

A self-report questionnaire and existing validated scales, which were suitable to the study purpose were utilized for data collection. Demographic data regarding participants’ age, professional experience, and experience in Group 1 games were self-reported. Physical-Self Description Questionnaire [23] adapted by Kim [24] was used to assess physical self-concept; it has 10 subscales (sports competence, body fat, appearance, health, physical activity, self-esteem, flexibility, endurance, muscle strength, and overall body). Similarly, occupational instability was assessed using the Job Insecurity Scale, which was developed by Ashford et al. [25] and modified by Kim and Ji [26]; it comprises three factors: occupation anxiety, duty anxiety, and personal anxiety. Lastly, retirement intention was measured using the Retirement Measurement Tool developed by Chae and Hong [27], which has nine sub-dimensions (addiction, faith and will, career choice, popularity climax, family support, injury or surgery, competitiveness, incidents, and self-esteem). The questionnaires were partially modified according to the purpose of the study; items were answered using a 5-point Likert scale, ranging from 1 (*not at all*) to 5 (*absolutely*). Additionally, the study variables of physical self-concept, occupational instability, and retirement intention were validated by a group of experts (two professors and five researchers with a Ph.D. in sports sociology) on a closed 2-point scale to secure content validity.

### 2.3. Reliability and Validity of the Instruments

Reliability refers to the degree to which the data indicate a specific object, and it is a criterion for evaluating whether there is a consistent and reproducible measurement [28]. In this study, Cronbach’s α was used to verify the internal consistency between the items of the test. The results are presented in Table 2. Cronbach’s α of the items measuring each latent variable ranged between 0.914–0.949. Items with a Cronbach’s α > 0.600 are considered appropriate [28]; therefore, the measures for latent variables in this study can be considered to have sufficient reliability.

Confirmatory factor analysis (CFA) is a method that analyzes all variables simultaneously and by classifying each concept [29]. Since there was sufficient evidence to establish a hypothetical model, this study only performed a CFA to verify the validity of the structural relationship between the variables simultaneously. To test the fit of the CFA, Chi-Square/Degrees of Freedom, Root Mean Square Residual, Comparative Fit Index (CFI), Incremental Fit Index, and Root Mean Square Error of Approximation (RMSEA) were used; the CFA fit of the proposed model is presented in Table 3. The model fit was found to be good. The criteria of CFI (>0.900) and RMSEA (<1.000) was used to determine the model fit suitability [30]; therefore, goodness-of-fit can be considered acceptable.

Moreover, the CFA results were used to verify convergent, discriminant, and legal validity. The criteria of standardized regression coefficient of all variables (>0.500), average variance ejection of latent variables (AVE; >0.600), and construct reliability (CR; >0.700), as prescribed by Anderson and Gerbing [31], was used to evaluate the degree of validity. Convergent validity was verified accordingly (Table 4). The standardized regression coefficients were 0.5 or more, and the significance (critical ratio) was 1.965 or more. Additionally, the CR was 0.971–0.993, and the AVE was 0.918–0.938, which satisfied all three criteria for establishing convergent validity.

Additionally, in this study, the discriminant validity was verified by comparing the correlation between the constituent concepts, which is the most rigorous method of verifying discriminant validity, along with the mean variance eruption [31]; the results are presented in Table 5. The squared correlation coefficient was highest (*r*^2^ = 0.524) between “physical self-concept” and “occupational instability”, which was lower than their individual AVE (physical self-concept = 0.938; occupational instability = 0.922). Therefore, the discriminant validity was sufficiently established. Finally, the legal validity was verified. In this study, the relationships between “physical self-concept ↔ occupational instability” and “physical self-concept ↔ retirement intention” were predicted in the negative (−) direction. However, the relationship between “occupational instability ↔ retirement intention” was predicted in the positive (+) direction, and according to Table 5, the predicted and observed direction were the same; thus, the law validity was adequately verified.

### 2.4. Procedure and Data Analysis

Data were analyzed using SPSS and AMOS 18.0 (IBM Corp., Armonk, NY, USA) software. A frequency analysis was performed to examine participants’ demographic characteristics. Further, the instruments used in this study were psychometrically validated using Cronbach’s α (reliability) and CFA (convergent, discriminatory, and law validity). Lastly, descriptive statistics and a path analysis were used to verify the fit of the hypothesis model to the structural relationship of each variable. The statistical significance was set at *p* < 0.05.

## 3. Results

### 3.1. Descriptive Statistics

The mean, standard deviation, skewness, and kurtosis of all latent and observed variables were computed (see Table 6). The mean values for all variables range between 1.69 and 3.28 (±0.66–1.11). Moreover, the absolute values of skewness and kurtosis range from 0.07–1.54 and 0.02–3.23, respectively. The univariate normality criteria of skewness (<±3.0) and kurtosis (<±10.0) were satisfied [32,33]. Thus, it can be assumed that the maximum likelihood estimation method does not affect the result, and it satisfies the structural model verification criteria [32,33].

### 3.2. Path Analysis

The fit of the hypothesized model was analyzed before verifying the research hypotheses of this study. Several researchers have reported various methods for verifying the suitability of a structural equation model [28,29]. For this purpose, verification of the hypothesis model fit should comprehensively assess various fit levels. In this study, the degree of fit was assessed using CFI (>0.900) and RMSEA (<0.100), as prescribed by Hong [30]. CFI represents the incremental fit index; thus, values closer to 1.00 indicate more appropriate models. On the other hand, RMSEA complements the thresholds of chi-squared distribution/degree of freedom, since they are sensitive to sample size and properties, and acts as an alternative to consider the model suitable, if the RMSEA value is less than 0.10, as has been widely implemented in recent studies. Thus, we used the criteria recommended by Hong [30] for determining the goodness of fit of the structural model because it can be considered the most effective method for verifying the hypothesized model, thereby, serving the purpose of this study. Therefore, based on these criteria, the hypothesized model-fit (CFI = 0.902, RMSEA = 0.087) was considered acceptable.

Table 7 presents the direct effect and properties of each variable on the structural relationship of the verified hypothesized model. The results suggested that physical self-concept had a significant negative effect (path coefficient = −0.870, *t* = −11.480, *p* < 0.001) on occupational instability among professional baseball players in the minor league, which confirmed Hypothesis 1. Furthermore, it was found that the effect of physical self-concept on retirement intention was not significant among professional baseball players in the minor league; thus, Hypothesis 2 was rejected. Lastly, the results reported that occupational instability had a significant negative effect (path coefficient = 0.987, *t* = 4.591, *p* < 0.001) on the retirement intentions of minor league professional baseball players, which confirmed Hypothesis 3.

## 4. Discussion

This study aimed to provide a theoretical basis for developing a realistic plan to improve the treatment of South Korean minor league players, by examining the structural relationship between physical self-concept, occupational instability, and retirement intention among second-team professional baseball players.

The study findings revealed that physical self-concept had a negative effect on occupational instability among minor league players in South Korea. There is no existing literature on the relationship between physical self-concept and occupational instability in athletes. However, it can be seen that job insecurity among professional sports players is related to their perceived physical ability, which can be directly linked to their ability and salary. Additionally, Alfermann [34] stated that to maximize the effect of exercise on physical self-concept: (1) exercise ability should be substantially improved through training, and (2) performance perception should be improved. It is difficult to be recognized in South Korea’s professional baseball world. Therefore, physical self-concept acts as an important variable to indicate a player’s skills and their existential value.

Moreover, the findings reported that physical self-concept of minor league players did not affect their retirement intentions directly, but through occupational instability. Previous studies [10,11,24] have reported that excellent athletes have higher physical self-concept than non-excellent athletes, which leads to higher skill development among the former with the same amount of effort as the latter. These results are not directly linked to retirement intentions because of players’ low physical self-concept, but as professional athletes, their ability to retire is lowered with the stability of the job, which is evident through a relative comparison with other athletes. All the second-team professional baseball players were more talented than other amateur players; their proficiency can be deduced from their eligibility to become professional players, or their career would have ended in middle and high school [1]; these players focused solely on baseball to achieve their dreams of becoming excellent professional baseball players. However, South Korean professional baseball—having the best baseball players from South Korea—is structured such that only those players can survive, who significantly improve their skills since their amateur years. Thus, a modified plan is required by the association and club to reinforce the physical self-concept of second-team players, for improving the skills and psychological stability of those who are unable to advance to the first team easily.

Another study finding revealed that occupational instability in minor league players had a positive effect on their retirement intentions. These findings are consistent with previous studies reporting that occupational instability is linked to retirement [19,20]. Professionals prove their existence and worth through their annual salary. According to the data released by the KBO [35], the average annual salary of 10 KBO league players in 2016, excluding rookies and foreigners, was about USD 28,000. However, only the statistical average has increased than before, the average experienced by the players has not increased. This statistical increase in average annual salary has occurred owing to the emergence of high-paid salaries incurred by very limited South Korean professional baseball players, who have a total free agent contract of more than USD 1,000,000. However, the minimum annual salary of a South Korean professional baseball player, including those struggling in no-attention games, is about USD 24,000. The minimum salary is lower than the starting salary of a general salaried employee, but it accounts for the player’s equipment and lifestyle costs. Thus, the salary gap between the first- and second-team players widens every year, and the polarization intensifies. Despite the long-term recognition of professional baseball as a national sport, with an average of 9 million spectators, the benefits are not enjoyed evenly by the second-team players. Motivating star players by providing higher salaries, such as free agent players, is beneficial, but it increases the salary gap and the relative deprivation experienced by second-team players [1]. Thus, the KBO and other clubs need to implement a practical raise and adjust the minimum salary of professional baseball players. The Korea Professional Baseball Council, which was established to safeguard the rights and interests of players, plays an important role, for this purpose, to prepare a device for high-paid players; however, it is necessary to establish a supportive atmosphere and realistic action plan to improve the treatment of the second-team players. Various measures should be implemented to support athletes, who have to retire involuntarily to allow them to adapt to the society comfortably post-retirement through further education and employment.

The results of this study had several implications: (1) To the best of our knowledge, previous studies have only verified the effect of these variables (physical self-concept, occupational instability, and retirement intention) sporadically and this is the first study reporting the structural relationship between these variables among minor league professional baseball players. (2) This study elucidates the important role of physical self-concept as a leading variable to reduce job insecurity and retirement intentions of second-team professional baseball players. (3) This study recognizes the struggle of players, who work hard in places that are overshadowed by the bright-looking professional baseball players, and it provides various institutional measures to improve their treatment and the urgent need to establish social stability post-retirement. This is an individual problem, which can lead to social problems; thus, it is necessary to prepare various measures to resolve it, using the theoretical basis provided in this study.

However, this study also had a few limitations: First, results were derived using quantitative research methods. This restricted the inclusion of stories with concrete meanings and contexts, which could not be objectively quantified. Future research should employ mixed methods to further analyze the difficulties and efforts of second-team baseball players. Second, this study examined only two variables—physical self-concept and occupational instability—to derive their effect on retirement intentions. However, there can be several other causes for retirement among minor league players; therefore, it is necessary to identify and analyze other variables influencing retirement in future studies. Third, the inclusion criteria restricted the recruitment of a sizable sample. Future studies need to expand the sample size further. Additionally, players, who are not professional baseball players but strive to succeed in the independent leagues can also be included in future studies. For this purpose, it will be necessary to target baseball players active in the independent league, in addition to the second-team players.

Following are a few suggestions for future research studies, specifically in order to tackle the uncontrolled and difficult avenues that we encountered during the current research. First, we derived meaningful results in this study by employing quantitative research methods. However, these methods were limited in their procedures for revealing specific meanings and contexts. It is necessary for further studies to examine the difficulties and efforts of minor league baseball players through mixed or qualitative research methods. Second, this study established a meaningful association of physical self-concept and occupational instability with retirement intention. However, various causes can lead to retirement for minor league baseball players; thus, future studies should identify and analyze various variables that cause retirement. Third, it was very difficult to obtain a large sample in this study due to the small population of minor league baseball players. Therefore, future research should focus on expanding the sample to minor league players of other countries/territories with active professional baseball, such as the United States, Japan, and Taiwan, as well as Korea, to examine the differences and commonalities between diverse samples of different countries/territories.

## 5. Conclusions

This study examined the structural relationship between physical self-concept, occupational instability, and retirement intentions of professional minor league baseball players. The results revealed that (1) physical self-concept of minor league players had a negative effect on occupational instability, (2) physical self-concept of minor league players did not affect their retirement intention, and (3) occupational instability in minor league players had a positive effect on retirement intentions. These results indicate the poor treatment of second-team professional baseball players and suggest the urgent need for bridging the extreme salary gap between first- and second-team professional baseball players.

## Figures and Tables

**Table 1:** Demographic characteristics of the participants.

Characteristics	Groups	*n*	%
Age (years)	20–25	84	46.7
	26–30	69	38.3
	Over 31	27	15.0
Professional experience (years)	5 years or less	89	49.5
	6–10 years	58	32.2
	10 years or more	33	18.3
Experience in second team (number of games played)	None	27	15.0
	50 games or less	53	29.4
	51–100 games	46	25.6
	101–200 games	31	17.2
	201 games or more	23	12.8
Total		180	100.0

Tested using frequency analysis.

**Table 2:** Verification of reliability.

Variables	Cronbach’s α
Physical self-concept	0.949
Occupational instability	0.914
Retirement intention	0.938

Tested using reliability analysis.

**Table 3:** Confirmatory factor analysis for the proposed model, verification of goodness-of-fit.

Goodness-of-Fit	Hypothetical Model
Chi-square/Degrees of freedom	2.368
Root mean square residual	0.074
Comparative fit index	0.902
Incremental fit index	0.902
Root mean square error of approximation	0.087

**Table 4:** Confirmatory factor analysis results.

Variables	Sub-Dimensions	Standardized Regression Coefficient	Non-Standardized Regression Coefficient	Standard Error	Critical Ratio	*p*	Construct Reliability	Average Variance Extracted
Physical self-concept	Sport competence	0.851	1.000	-	-	-	0.993	0.938
	Body fat	0.832	0.952	0.066	14.32	<0.001		
	Appearance	0.841	0.983	0.067	14.59	<0.001		
	Health	0.788	0.920	0.070	13.10	<0.001		
	Physical activity	0.838	1.030	0.071	14.52	<0.001		
	Self-respect	0.881	1.081	0.068	15.90	<0.001		
	Flexibility	0.763	0.895	0.072	12.44	<0.001		
	Endurance	0.684	0.717	0.068	10.60	<0.001		
	Muscle strength	0.824	0.931	0.066	14.11	<0.001		
	Overall body	0.761	0.878	0.071	12.40	<0.001		
Occupational instability	Personal anxiety	0.917	1.000	-	-	-	0.971	0.922
	Job anxiety	0.725	0.862	0.074	11.70	<0.001		
	Occupation anxiety	0.539	0.559	0.072	7.76	<0.001		
Retirement intention	Addiction	0.815	1.000	-	-	-	0.990	0.918
	Faith and will	0.851	0.950	0.071	13.31	<0.001		
	Career choice	0.715	0.977	0.093	10.50	<0.001		
	Popularity climax	0.732	0.849	0.078	10.83	<0.001		
	Family support	0.670	0.913	0.094	9.66	<0.001		
	Injury or surgery	0.705	1.061	0.103	10.31	<0.001		
	Competitiveness	0.654	0.957	0.102	9.38	<0.001		
	Incident	0.670	0.799	0.083	9.66	<0.001		
	Self-esteem	0.605	0.792	0.093	8.525	<0.001		

Tested using Confirmatory factor analysis.

**Table 5:** Correlation between constituent concepts.

Scale	Correlation between Constituent Concepts		
	Physical Self-Concept	Occupational Instability	Retirement Intention
Physical self-concept	1.000	-	-
Occupational instability	−0.724 ***	1.000	-
Retirement intention	−0.524 ***	0.690 ***	1.000
Average variance eruption	0.938	0.922	0.918

*** *p* < 0.001, tested using correlation analysis.

**Table 6:** Descriptive statistics.

Variables	Mean	Standard Deviation	Skewness	Kurtosis
Sport competence	3.07	1.06	0.20	−0.54
Body fat	2.93	1.03	0.24	−0.37
Appearance	2.82	1.05	0.16	−0.63
Health	2.88	1.05	0.19	−0.38
Physical activity	2.71	1.11	0.49	−0.38
Self-respect	3.28	1.10	−0.12	−0.75
Flexibility	2.79	1.06	0.19	−0.50
Endurance	2.29	0.94	0.47	−0.10
Muscle strength	2.51	1.02	0.23	−0.59
Overall body	2.32	1.04	0.58	−0.14
Physical self-concept	2.76	0.87	0.16	−0.38
Personal anxiety	3.14	0.90	−0.15	−0.51
Job anxiety	3.15	0.98	−0.28	−0.44
Occupation anxiety	2.19	0.85	0.74	0.71
Occupational instability	2.83	0.75	−0.07	−0.28
Addiction	2.28	0.84	0.52	0.28
Faith and will	2.34	0.76	0.74	1.41
Career choice	2.57	0.93	0.31	−0.32
Popularity climax	1.78	0.79	1.26	1.77
Family support	2.46	0.93	0.60	0.74
Injury or surgery	2.34	1.03	0.68	0.21
Competitiveness	2.23	1.00	0.57	0.02
Incident	1.69	0.81	1.54	3.23
Self-esteem	2.03	0.89	0.71	0.33
Retirement intention	2.19	0.66	1.02	2.60

Tested using descriptive statistical analysis.

**Table 7:** Result of the path analysis.

Path				Standardized Regression Coefficient	Non-Standardized Regression Coefficient	Standard Error	Critical Ratio	*p*	
H1	A	→	B	−0.870	−0.906	0.079	−11.480	<0.001	accepted
H2	A	→	C	0.280	0.242	0.168	1.444	0.149	rejected
H3	B	→	C	0.987	0.818	0.178	4.591	<0.001	accepted

A = physical self-concept, B = occupational instability, C = retirement intention. Tested using path analysis.

## Data Availability

The data presented in this study are available on request to the authors.
